# Inadequate unit selection for diffuse reflectance FT-IR spectra conceals low intensity bands of adsorbates

**DOI:** 10.1039/d6ra00308g

**Published:** 2026-02-11

**Authors:** F. C. Meunier

**Affiliations:** a Université Lyon 1, CNRS, UMR 5256, IRCELYON, Institut de Recherches sur La Catalyse et L’Environnement de Lyon 2 Avenue Albert Einstein F-69626 Villeurbanne France fcm@ircelyon.univ-lyon1.fr

## Abstract

Diffuse Reflectance FT-IR spectroscopy (DRIFTS) is a prominent tool to investigate adsorbates on catalyst surfaces. Low intensity bands of adsorbates are inappropriately squashed when using the Kubelka–Munk transformation, concealing the Pd–CO signal associated with Pd nanoparticles in the example reported here.

## Abstract 2

The Kubelka–Munk transformation squashes low intensity DRIFTS bands, misrepresenting site speciation.

## Graphical abstract

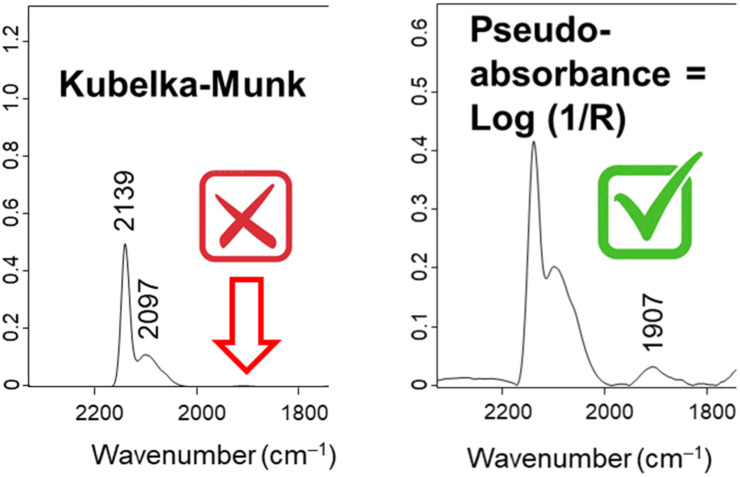

Diffuse reflectance FT-IR spectroscopy (DRIFTS) is used to investigate heterogeneous catalysts under reaction conditions^1–3^ and characterise surface sites through the adsorption of probe molecules such as CO.^4–7^ Kubelka–Munk (eqn (1)) and pseudo-absorbance (eqn (2)) transformations are both used to report adsorbate spectra:

1 Kulbelka–Munk: *f*(*R*_∞_) = (1 − *R*_∞_)^2^/(2*R*_∞_)

2 Pseudo-absorbance: Abs (*R*_∞_) = Log (1/*R*_∞_)

where *R*_∞_ is the absolute reflectance of the scattered radiation (scattered intensity divided by that of the incident radiation). Note that measuring *R*_∞_ would require using an integration sphere, which is rarely the case, hence only a fraction of *R*_∞_ is actually measured.

It has yet been shown that the pseudo-absorbance is a more appropriate transformation when the intensity of adsorbate bands is low.^8,9^ Pseudo-absorbance shows a better proportionality to adsorbate surface coverage, while Kubelka–Munk units were shown to underrepresent the intensity of minor bands. The transformation that actually represents best surface coverage is proportional to the so-called Matyshak–Krylov transformation (eqn (3)):

3 Matyshak–Krylov(*R*,*R*_∞_) = (*R*_∞_ − *R*)(1/*R* − *R*_∞_)/*R*_∞_

where *R*_∞_ and *R* are the catalyst and the (catalyst + adsorbate) reflectance relative to a reference sample, respectively.^8,10^

CO adsorption is commonly used to investigate the nature of metal sites on catalysts, although spectral analysis is not straightforward.^4,5^ A recent publication by Han and co-workers reported on Pd single atoms and Pd nanoparticles supported on ceria and characterised by DRIFTS of CO adsorption.^11,12^ A Kubelka–Munk spectrum of a 5 wt% Pd/CeO_2_ oxidised at 550 °C was kindly supplied to us by Prof Han and is shown in Fig. 1a.

**Fig. 1:**
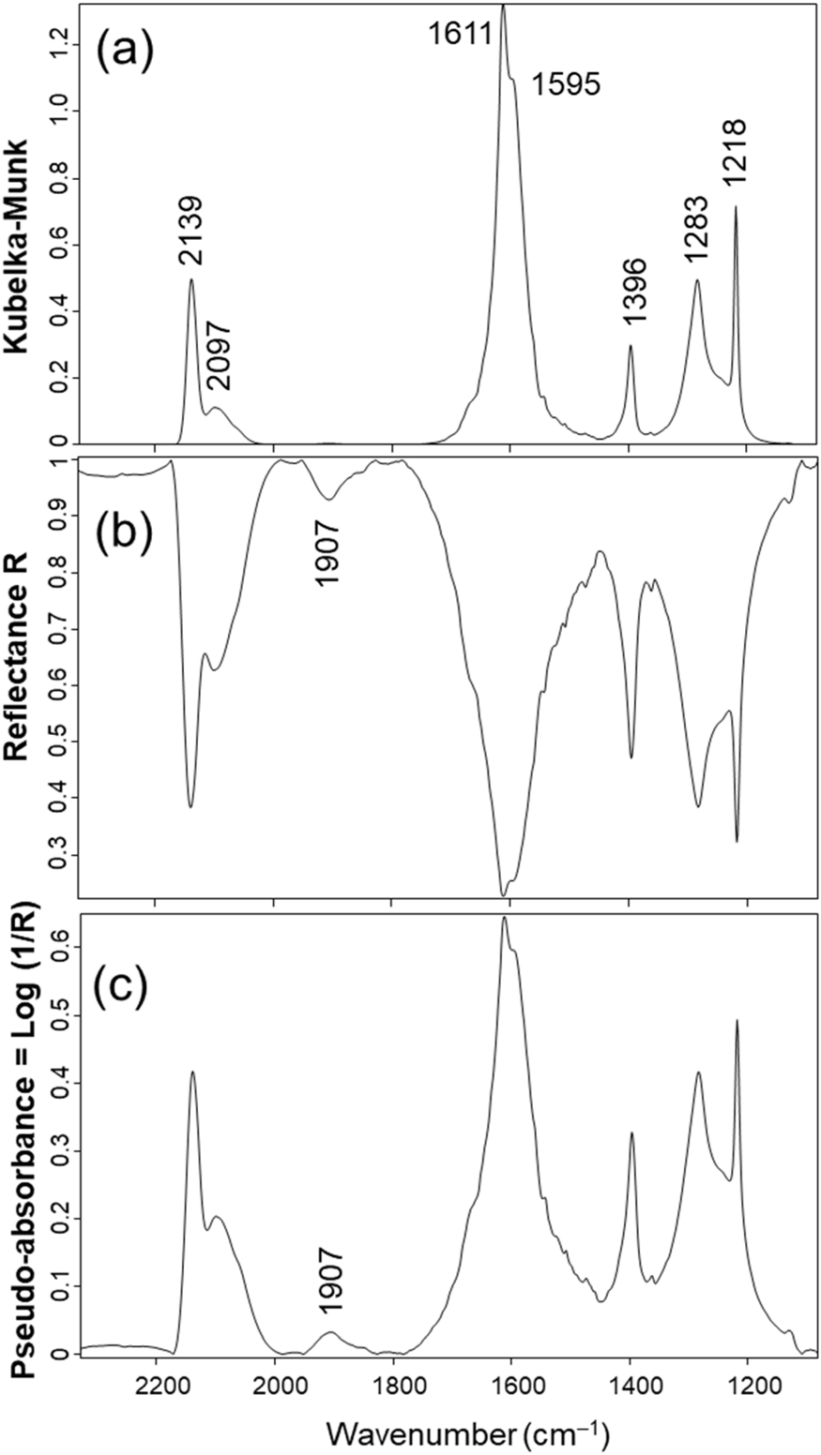
DRIFTS spectrum of the Pt/CeO_2_-Ox-550 sample exposed to 2 mbar of carbon monoxide reported as (a) Kubelka–Munk units, (b) corresponding reflectance and (c) corresponding pseudo-absorbance.

The position of the Pd–CO bands of interest are consistent with those reported earlier:^11^ a main sharp band at 2139 cm^−1^ and a broader one at 2097 cm^−1^ that can be assigned to CO adsorbed on isolated Pd cations on ceria.^6,7^ The absence of band between 2000 and 1800 cm^−1^ indicates that no CO adsorbed on metallic Pd nanoparticles could be detected.^13^ The other observable bands below 1800 cm^−1^ were due to hydrogenocarbonates (1611, 1396 and 1218 cm^−1^) and carbonates (1595 and 1283 cm^−1^) adsorbed on ceria.^15^

The corresponding reflectance was computed by the OPUS software and is shown in Fig. 1b. Interestingly, a band at 1907 cm^−1^ could then be observed, which can be assigned to CO adsorbed on metallic nanoparticles.^13,14^ The intensity of this band was weak, exhibiting a reflectance higher than 90% (Fig. 1b). A further conversion of the reflectance spectrum to the pseudo-absorbance transformation was made (Fig. 1c). It is interesting to note that the pseudo-absorbance spectrum was similar to that of Kubelka–Munk, except for the appearance of the 1907 cm^−1^ band associated with CO adsorbed on Pd nanoparticles.

These data stress that bands exhibiting a small intensity (reflectance higher than 90% in the present case, Fig. 1b) do not lead to any observable feature when reported in Kubelka–Munk units (Fig. 1a). In contrast, these bands can be observed when reporting the pseudo-absorbance transformation (Fig. 1c).

The fact that Kubelka–Munk concealed weak bands has been discussed earlier (see Fig. 7 in ref. 8) and is again illustrated below. The values of the Matyshak–Krylov, Kubelka–Munk and pseudo-absorbance functions were calculated for a reflectance *R*′ varying from 1 to 0.8, where *R*′ was the ratio of the reflectance *R* measured over the catalyst covered with adsorbate and *R*_∞_, which is that of the pristine catalyst (Table 1).

**Table 1:** Hypothetical values of intensities (*I*_o_ incident, *I*_cat_ diffused by the catalyst and *I*_cat+ads_ diffused by the catalyst covered with adsorbate) and calculated reflectance (*R*_∞_ from the catalyst, *R* from the catalyst covered with adsorbate and relative *R*′ = *R*/*R*_∞_), pseudo-absorbance Log(1/*R*′), Kubelka–Munk(*R*′) and Matyshak–Krylov(*R*,*R*_∞_) functions

*I*_0_	*I*_cat_	*I*_cat+ads_	*R*_∞_	*R*	*R*′ = *R*/*R*_∞_	Log (1/*R*′)	Kubelka–Munk(*R*′)	Matyshak–Krylov(*R*,*R*_∞_)
1	0.5	0.500	0.5	0.500	1.00	0.0000	0.0000	0.00
1	0.5	0.495	0.5	0.495	0.99	0.0044	0.0001	0.02
1	0.5	0.490	0.5	0.490	0.98	0.0088	0.0002	0.03
1	0.5	0.485	0.5	0.485	0.97	0.0132	0.0005	0.05
1	0.5	0.480	0.5	0.480	0.96	0.0177	0.0008	0.06
1	0.5	0.475	0.5	0.475	0.95	0.0223	0.0013	0.08
1	0.5	0.470	0.5	0.470	0.94	0.0269	0.0019	0.10
1	0.5	0.465	0.5	0.465	0.93	0.0315	0.0026	0.12
1	0.5	0.460	0.5	0.460	0.92	0.0362	0.0035	0.13
1	0.5	0.455	0.5	0.455	0.91	0.0410	0.0045	0.15
1	0.5	0.450	0.5	0.450	0.90	0.0458	0.0056	0.17
1	0.5	0.445	0.5	0.445	0.89	0.0506	0.0068	0.19
1	0.5	0.440	0.5	0.440	0.88	0.0555	0.0082	0.21
1	0.5	0.435	0.5	0.435	0.87	0.0605	0.0097	0.23
1	0.5	0.430	0.5	0.430	0.86	0.0655	0.0114	0.26
1	0.5	0.425	0.5	0.425	0.85	0.0706	0.0132	0.28
1	0.5	0.420	0.5	0.420	0.84	0.0757	0.0152	0.30
1	0.5	0.415	0.5	0.415	0.83	0.0809	0.0174	0.32
1	0.5	0.410	0.5	0.410	0.82	0.0862	0.0198	0.35
1	0.5	0.405	0.5	0.405	0.81	0.0915	0.0223	0.37
1	0.5	0.400	0.5	0.400	0.80	0.0969	0.0250	0.40

The plot of pseudo-absorbance *versus* the Matysha–Krylov function (which is proportional to adsorbate surface concentration^8,10^) was essentially linear, indicating the relevance of pseudo-absorbance to represent adsorbate concentration (Fig. 2). In contrast, the plot of the Kubelka–Munk function was a curve with a zero-slope tangent at the origin.

**Fig. 2:**
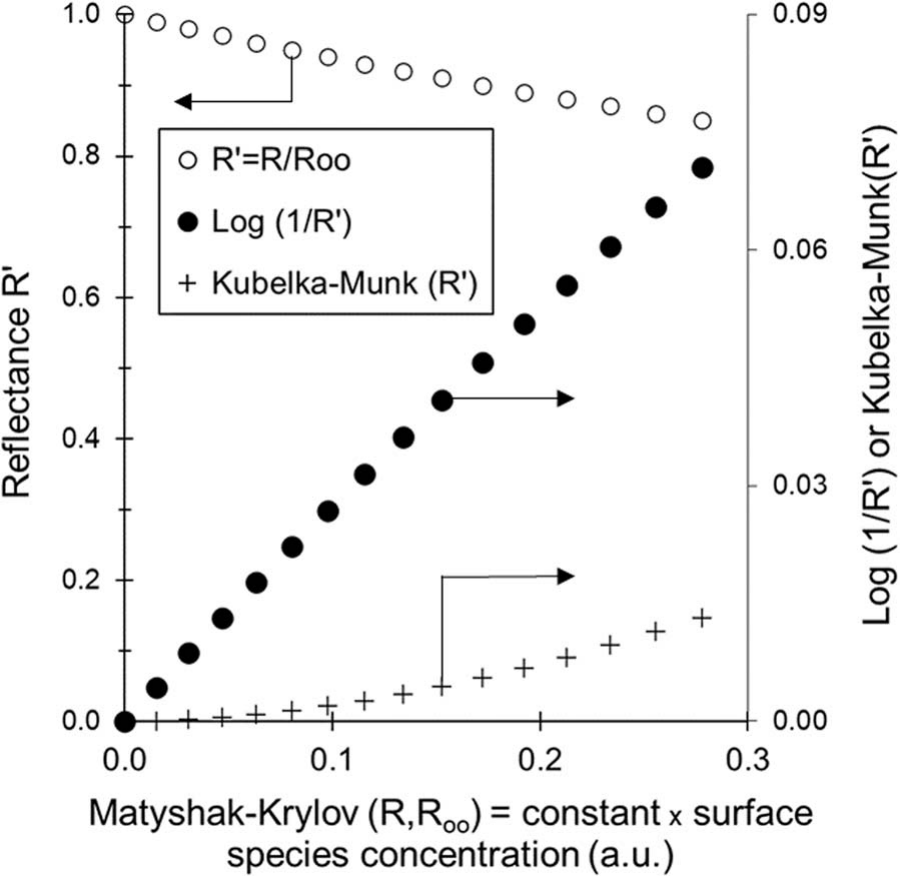
Values of reflectance *R*′, pseudo-absorbance Log(1/*R*′) and Kubelka–Munk(*R*′) transformations as a function of the Matyshak–Krylov(*R*,*R*_∞_) function for adsorbate weak band intensity, *i.e. R*′ higher than 80%. The datapoints are reported in Table 2.

This indicates that weak bands are essentially squashed by the Kubelka–Munk function, giving a biased picture of the proportions of species present at the surface. This is especially important over the present catalyst that actually exhibits both bands associated with Pd single cations (bands at 2139 and 2097 cm^−1^) and Pd metallic nanoparticles (band at 1907 cm^−1^)(Fig. 1c). Metallic nanoparticles have been shown to exhibits catalytic activities four orders of magnitude higher than that of the corresponding single atoms in the case of CO oxidation over Pt/CeO_2_.^3,16^ Therefore, there is a genuine risk that catalytic activity may be assigned to the wrong surface sites, if weak bands are mathematically erased.

## Conclusions

The utilization of the Kulbelka–Munk function when reporting diffuse reflectance FT-IR spectra essentially erases bands with weak intensities (reflectance *R*′ > 90%) that may correspond to sites crucial for the catalytic properties of the material. It is thus recommended to report DRIFTS data of adsorbates as pseudo-absorbance = Log (1/*R*′).

## Conflicts of interest

There are no conflicts to declare.

## Data Availability

Data will be made available upon request to the corresponding author.
